# Heterotopic Cesarean Scar Pregnancy: A Case Report

**DOI:** 10.7759/cureus.55943

**Published:** 2024-03-11

**Authors:** Mariam Tariq Ahmed Alabsi, Amala Sunder, Abeer AlSada

**Affiliations:** 1 Obstetrics and Gynaecology, Bahrain Defence Force Hospital, Riffa, BHR

**Keywords:** outcome, management, diagnosis, ectopic pregnancy, extrauterine, heterotopic

## Abstract

Heterotopic pregnancy is a rare condition with an intrauterine pregnancy coexisting with extrauterine pregnancy. It is considered a rare condition, with an incidence of one in 30,000 spontaneous pregnancies. Because of its rarity, there are no international management guidelines. It is associated with increased maternal morbidity and mortality. Heterotopic pregnancy is a challenging condition to diagnose because it can be missed due to the intrauterine pregnancy. The most common signs and symptoms to support and help us reach a diagnosis would be adnexal mass, abdominal pain, and enlarged uterus.

In this case report, we aim to compare the clinical course and the outcome of intrauterine gestation while coexisting with scar pregnancy and the management options. This case report concerns a 34-year-old pregnant woman with a heterotopic pregnancy managed in Bahrain Defence Force Hospital.

## Introduction

Heterotopic pregnancy is a rare condition with an intrauterine pregnancy coexisting with extrauterine pregnancy [1]. The word heterotopic arises from the Greek language, in which “hetero” means other and “topos” means place [2]. Heterotopic pregnancy implies that two or more implantation sites co-occur. This is rare, although it is common in women with assisted reproductive techniques. It is a life-threatening condition in which both intrauterine pregnancy and extrauterine pregnancy as an ectopic coexist [3], in which both pregnancies are viable, with an incidence of one in 30,000 pregnancies [4,5]. The most common signs and symptoms that aid in reaching a diagnosis are adnexal mass, abdominal pain, and an enlarged uterus [6]. The management options pose a risk as it implies terminating the extrauterine pregnancy while retaining the intrauterine pregnancy [7]. This case reports a 34-year-old pregnant woman who presented with a heterotopic pregnancy that was managed conservatively.

## Case presentation

We present a rare case of heterotopic pregnancy with live intrauterine gestation and scar pregnancy [8]. A 34-year-old female (gravida 4 para 3) presented to the outpatient clinic on her first visit on May 8, 2022, complaining of a delayed period. Her last menstrual period was on March 26, 2022. Her past obstetric history was as follows: she had a lower segment cesarean section due to failed induction of labor in 2018; previous to that she had two full-term normal vaginal deliveries. At the booking visit, transvaginal sonography (TVS) showed a single intrauterine gestational sac (0.69 cm), a fetal pole was seen, and crown-rump length (CRL) was five weeks + five days, but fetal pulsation was not seen.

**Figure 1:**
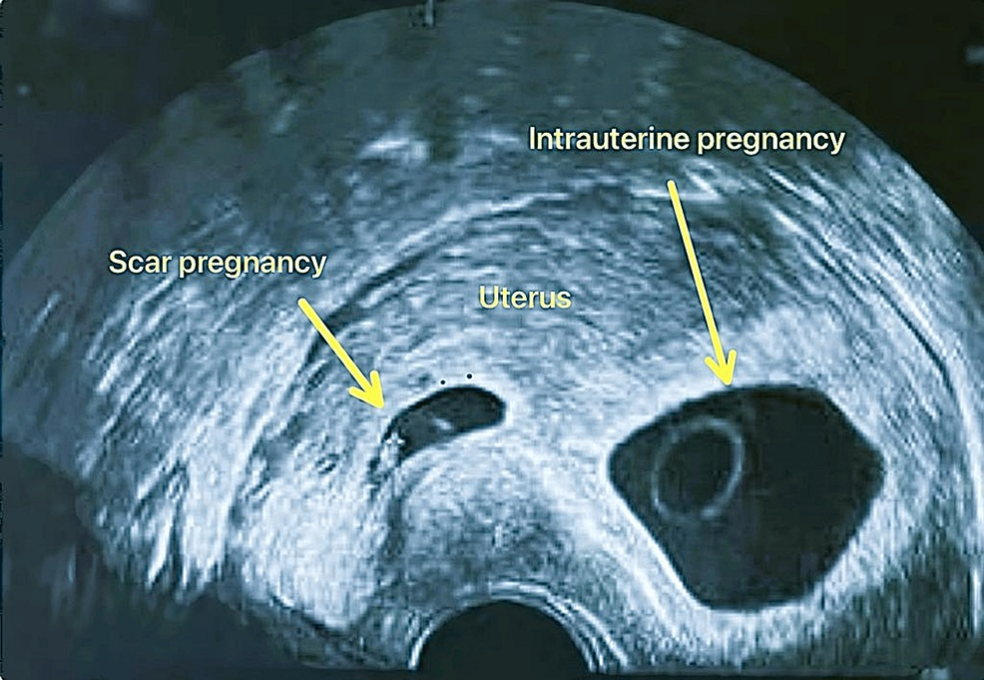
A scar of the previous lower segment cesarean section and the pregnancy embedded in it.

On the patient's second visit, TVS showed two gestational sacs: the first intrauterine gestational sac with fetal pole and fetal pulsation corresponding to seven weeks + two days and the second gestational sac with the fetus in the lower segment near the scar with CRL of six weeks + two days and no cardiac pulsations (Figure 1).

The patient was advised to rescan and follow up. Counseling was done about the option of transvaginal ultrasound-guided instillation of methotrexate into the scar pregnancy, and it may affect her normal intrauterine fetus that may end with miscarriage. The patient refused the option of using methotrexate.

Upon her third visit, TVS revealed that T1 corresponded to eight weeks + one day with fetal heart pulsation, while T2 had no fetal heart pulsation. The patient was advised for conservative treatment, and to come directly to the emergency department for any bleeding or pain. The patient was started on Proluton Depot 250 mg biweekly, Cyclogest pessary 400 mg once a day, and Duphaston 10 mg twice a day orally to support the viable pregnancy and conservative treatment for the scar pregnancy.

Eventually, the scar pregnancy vanished during her follow-up antenatal clinic visits.

The patient then continued regular antenatal follow-ups, and all visits were uneventful. On her gestational age (GA) of 37 weeks + four days, the patient was booked for her elective cesarean section at 38 weeks GA (as the patient requested). An ultrasound was done and showed a single active fetus, cephalic, with an estimated fetal weight of 2.8 kg, and Doppler was normal with the upper placenta. Her cesarean section was uneventful.

## Discussion

Heterotopic cesarean scar pregnancy is a rare and challenging condition due to the patient's desire to maintain and keep the intrauterine pregnancy. An attentive early scan is important whenever there is a history of cesarean section. Isolated cesarean scar pregnancy occurs with an incidence of one in 2000 [9]. The incidence of tubal ectopic pregnancy is also rising due to the rising risk of pelvic inflammatory disease, tubal damage that can be caused by assisted ovulation, induction management, use of intrauterine contraceptive devices, and tubal surgery [4,10].

It was noted in a systematic review that nearly a third of heterotopic pregnancies are diagnosed after spontaneous conception [11], and sometimes, diagnosis is not made until rupture happens, and hypovolemic shock occurs in heterotrophic ectopic pregnancies [3,4].

Early diagnosis of heterotopic pregnancy may help the preservation of the intrauterine pregnancy.

Our patient had a risk that exposed her to developing this condition; a previous cesarean section attributed to the scar pregnancy. She presented with a complaint of a delayed period (amenorrhea) with no other symptoms. TVS was done that revealed an intrauterine gestational sac with a fetal pole, which, after a few visits, showed that there was a scar pregnancy.

The management poses a significant risk because it aims to terminate the extrauterine pregnancy while retaining the intrauterine pregnancy [4]. The treatment options for cesarean scar pregnancy are methotrexate injection, potassium chloride injection, and transvaginal embryo aspiration [3].

The earlier selective intervention of the heterotopic scar pregnancy would be the goal for a better outcome [12].

Selective aspiration of the scar pregnancy is one of the acceptable management options to preserve the intrauterine viable fetus [13]. We added progesterone support for the viable intrauterine pregnancy to prevent early miscarriage [14].

Complications of residual pregnancy such as septic miscarriage are possible after selective reduction of scar pregnancy [15]. Following the selective reduction of massive bleeding, placentae percreta are to be encountered during pregnancy [16]. However, in our case, spontaneous resolution of the scar pregnancy leads the way for the successful outcome of the intrauterine pregnancy without the intervention of the heterotopic scar pregnancy.

In this case, the patient was counseled about transvaginal ultrasound-guided instillation of methotrexate into the scar pregnancy, which also can affect intrauterine pregnancy. The patient refused the management option of the scar pregnancy. She chose elective cesarean section for the normal intrauterine pregnancy, which was planned and performed with the optimized outcome.

## Conclusions

Heterotopic cesarean scar pregnancy is a rare type of heterotopic pregnancy. Adequate counseling of management options, including benefits and risks, is required. The presented case shows the successful preservation of intrauterine pregnancy in heterotrophic cesarean scar pregnancy as well as the spontaneous resolution of heterotopic pregnancy without complications of the intrauterine viable pregnancy. Though the case required adequate counseling of management options, she was not in need of intervention as the scar pregnancy resolved spontaneously without complications. Supervised and supportive monitoring of the intrauterine pregnancy provided the optimized term baby.
